# 8-{[(*E*)-3-(2-Chloro­phen­yl)acrylo­yloxy]imino}-12,13-ep­oxy­trichethec-9-en-4-yl (*E*)-3-(2-chloro­phen­yl)acrylate

**DOI:** 10.1107/S1600536811039638

**Published:** 2011-10-05

**Authors:** Xiao-Jun Xu, Xiao-Liang Li, Jing-li Cheng, Jin-hao Zhao

**Affiliations:** a College of Pharmaceutical Science, Zhejiang University of Technology, Hangzhou 310032, People’s Republic of China; b Department of Chemistry, Zhejiang Sci-Tech University, Hangzhou 310018, People’s Republic of China; c Institute of Pesticide and Environmental Toxicology, Zhejiang University, Hangzhou 310029, People’s Republic of China

## Abstract

In the title compound, C_33_H_31_Cl_2_NO_6_, the five-membered ring displays an envelope conformation, whereas the two six-membered rings both exhibit a chair conformation. As for the seven-membered ring, the dihedral angle between the mean planes formed by the four C atoms of the envelope unit and the three C and one O atoms of the six-membered chair is 69.08 (4)°, and these two mean planes are nearly perpendicular to the ep­oxy ring, making dihedral angles of 87.53 (4) and 88.67 (4)°, respectively.

## Related literature

The endophytic fungi Trichoderma taxi *sp*. nov. from Taxus mairei S. Y. Hu can produce a compound with fungicidal activity, Trichodermin (Zhang *et al.*, 2007 ▶), which is a member of the 4β-acet­oxy-12,13-ep­oxy­trichothecene family (Nielsen *et al.*, 2005 ▶). For a related Trichodermin structure, see: Chen *et al.* (2008 ▶). For structures of Trichodermin derivatives, see: Cheng *et al.* (2009 ▶); Xu *et al.* (2010 ▶).
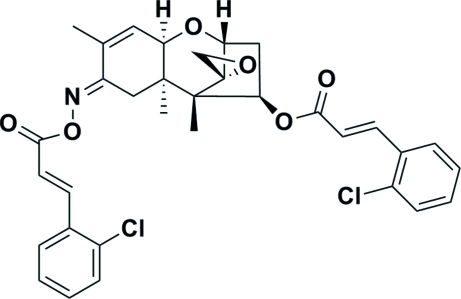


## Experimental

### 

#### Crystal data

C_33_H_31_Cl_2_NO_6_
*M* *_r_* = 608.49
Monoclinic, 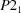

*a* = 7.2302 (4) Å
*b* = 14.4055 (6) Å
*c* = 14.6663 (6) Å
β = 94.414 (1)°
*V* = 1523.03 (12) Å^3^
*Z* = 2
Mo *K*α radiation
μ = 0.26 mm^−1^
*T* = 296 K
0.41 × 0.36 × 0.29 mm

#### Data collection

Rigaku R-AXIS RAPID/ZJUG diffractometer
Absorption correction: multi-scan (*ABSCOR*; Higashi, 1995 ▶) *T*_min_ = 0.895, *T*_max_ = 0.929
15059 measured reflections
6713 independent reflections
3890 reflections with *I* > 2σ(*I*)
*R*_int_ = 0.026

#### Refinement

*R*[*F* ^2^ > 2σ(*F* ^2^)] = 0.040
*wR*(*F* ^2^) = 0.130
*S* = 1.00
6713 reflections
384 parameters
7 restraints
H-atom parameters constrained
Δρ_max_ = 0.40 e Å^−3^
Δρ_min_ = −0.30 e Å^−3^
Absolute structure: Flack (1983 ▶), 3099 Friedel pairs
Flack parameter: 0.02 (8)

### 

Data collection: *PROCESS-AUTO* (Rigaku, 2006 ▶); cell refinement: *PROCESS-AUTO*; data reduction: *CrystalStructure* (Rigaku, 2007) ▶; program(s) used to solve structure: *SHELXS97* (Sheldrick, 2008 ▶); program(s) used to refine structure: *SHELXL97* (Sheldrick, 2008 ▶); molecular graphics: *ORTEP-3 for Windows* (Farrugia, 1997 ▶); software used to prepare material for publication: *WinGX* (Farrugia, 1999 ▶).

## Supplementary Material

Crystal structure: contains datablock(s) global, I. DOI: 10.1107/S1600536811039638/zq2124sup1.cif

Structure factors: contains datablock(s) I. DOI: 10.1107/S1600536811039638/zq2124Isup2.hkl

Additional supplementary materials:  crystallographic information; 3D view; checkCIF report

## Figures and Tables

**Fig. 1.**
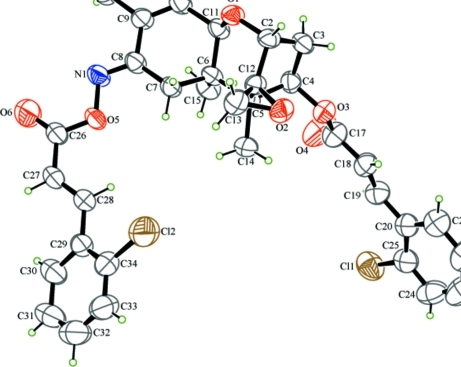
The molecular structure of (I) showing the atom-labelling scheme. Displacement ellipsoids are drawn at the 40% probability level.

**Fig. 2.**
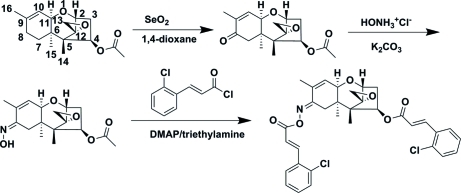
Reaction scheme.

## supplementary crystallographic information

### Comment

The endophytic fungi Trichoderma taxi *sp*. nov. from Taxus mairei S. Y. Hu can produce a compound with fungicidal activity - Trichodermin (Zhang *et al.*, 2007), which is a member of the 4β-acetoxy-12,13-epoxytrichothecene family (Nielsen *et al.*, 2005). Bioassays showed Trichodermin strongly inhibited Rhizoctonia solani and Botrytis cinere. In order to find the relationship between the conjugated double bonds at 8 and 4 positions and biological activities, we designed to take the esterification reaction, thus, the title compound had been synthesized. Its molecular structure is shown in Fig. 1. In the molecule, the five membered ring displays an envelope conformation with atom C12 at the flap position 0.694 (5) Å out of the mean plane formed by C2, C3, C4 and C5. The pyranyl ring displays a chair conformation with the C11 and C12 atoms deviating by -0.578 (5) and 0.843 (5) Å from the mean plane formed by O1, C2, C5 and C6. It is interesting to note that the C8–C9–C10 bond angle is smaller than in the structure of Trichodermin (Chen *et al.*, 2008) and Trichodermol (4α-hydroxy-12,13-epoxytrichothec-9-ene) (Cheng *et al.*, 2009), two hydrogen bonds being displaced by the presence of the carbon-nitrogen double bond in the title compound. As for the seven-membered ring, the dihedral angle between the mean planes formed by C2–C3–C4–C5 and C2–C5–C6–O1 is 69.08 (4) °, two planes which are nearly perpendicular to the three-membered ring with angles of 87.53 (4) and 88.67 (4)°, respectively.

### Experimental

In a flask, a solution of trichodermin (4.00 g, 13.70 mmoL) in 30 ml 1,4-dioxane was added dropwise over a period of 90 min to a refluxing solution of selenium dioxide (2.00 g, 18.02 mmoL) in 20 ml of 1,4-dioxane. After refluxing 12 h, the reaction was cooled and concentrated. Then 50 ml of ethyl acetate was added, and was washed with 5% aqueous sodium bicarbonate, dried, and concentrated in vacuum to 3.50 g of yellow liquid. The crude product was purified by flash column chromatography on silica gel using a mixture of petroleum ether and ethyl acetate (5: 1 by volume) as the eluent to give trichodermin-8-one (1.10 g, 26.2%) as colorless crystals. Then a mixture of trichodermin-8-one (1.00 g, 3.27 mmoL), hydroxylammonium chloride (0.45 g, 6.52 mmoL), and 30 ml methanol was stirred at 80 °C for 0.2 h. After the mixture was dissolved, a solution of potassium carbonate (4.00 g, 13.70 mmoL) in 20 ml water was added dropwise. The solution was stirred for 2.5 h, and concentrated, then extracted by ethyl acetate (10 ml) for three times, evaporated, and the crude product was purified *via* silica gel column chromatography using a 1: 2 (*v*/*v*) mixture of ethyl acetate and petroleum ether (boiling point range 60–90 °C) as the eluting solution to obtain (*E*)-trichodermin-8-hydroxy oxime as colorless crystals (0.89 g, 85.0%). At last, (*E*)-3-(2-chlorophenyl)acryloyl chloride (1.11 g, 5.54 mmoL) with 5 ml dichloromethane was added dropwise into a mixture of (*E*)-trichodermin-8-hydroxy oxime (0.89 g, 2.77 mmoL), triethylamine (33.8 mg, 0.28 mmoL), and *N*,*N*-dimethylpyridin-4-amine (0.56 g, 5.54 mmoL). The solution was stirred at room temperature and monitored by TLC. After 0.5 h, the mixture was washed with 1 N HCl, sat. NaHCO_3_ and dried over anhydrous Na_2_SO_4_. The solvent was evaporated *in vacuo* to afford the crude product, which was purified by column chromatography to give the title compound (1.35 g, 80%) as a colorless solid. The solid was filtrated and recrystallized with acetone to get colourless blocks.

### Refinement

The H atoms were geometrically placed and refined as riding, with C–H = 0.93 Å and *U*_iso_(H) = 1.2*U*_eq_(C) for aromatic H atoms, with C–H = 0.97 Å and *U*_iso_(H) = 1.2*U*_eq_(C) for methylene H atoms, with C–H = 0.98 Å and *U*_iso_(H) = 1.2*U*_eq_(C) for methine H atoms, and with C–H = 0.96 Å and *U*_iso_(H) = 1.5*U*_eq_(C) for methyl H atoms.

### Crystal data

**Table:**

C_33_H_31_Cl_2_NO_6_	*F*(000) = 636
*M**_r_* = 608.49	*D*_x_ = 1.327 Mg m^−^^3^
Monoclinic, *P*2_1_	Mo *K*α radiation, λ = 0.71073 Å
Hall symbol: P 2yb	Cell parameters from 9966 reflections
*a* = 7.2302 (4) Å	θ = 3.1–27.4°
*b* = 14.4055 (6) Å	µ = 0.26 mm^−^^1^
*c* = 14.6663 (6) Å	*T* = 296 K
β = 94.414 (1)°	Chunk, colourless
*V* = 1523.03 (12) Å^3^	0.41 × 0.36 × 0.29 mm
*Z* = 2	

### Data collection

**Table:**

Rigaku R-AXIS RAPID/ZJUG diffractometer	6713 independent reflections
Radiation source: rolling anode	3890 reflections with *I* > 2σ(*I*)
graphite	*R*_int_ = 0.026
Detector resolution: 10.00 pixels mm^-1^	θ_max_ = 27.4°, θ_min_ = 3.1°
ω scans	*h* = −9→9
Absorption correction: multi-scan (*ABSCOR*; Higashi, 1995)	*k* = −18→18
*T*_min_ = 0.895, *T*_max_ = 0.929	*l* = −18→18
15059 measured reflections	

### Refinement

**Table:**

Refinement on *F*^2^	Hydrogen site location: inferred from neighbouring sites
Least-squares matrix: full	H-atom parameters constrained
*R*[*F*^2^ > 2σ(*F*^2^)] = 0.040	*w* = 1/[σ^2^(*F*_o_^2^) + (0.0437*P*)^2^ + 0.8334*P*] where *P* = (*F*_o_^2^ + 2*F*_c_^2^)/3
*wR*(*F*^2^) = 0.130	(Δ/σ)_max_ < 0.001
*S* = 1.00	Δρ_max_ = 0.40 e Å^−^^3^
6713 reflections	Δρ_min_ = −0.30 e Å^−^^3^
384 parameters	Extinction correction: *SHELXL97* (Sheldrick, 2008), Fc^*^=kFc[1+0.001xFc^2^λ^3^/sin(2θ)]^-1/4^
7 restraints	Extinction coefficient: 0.0140 (15)
Primary atom site location: structure-invariant direct methods	Absolute structure: Flack (1983), 3099 Friedel pairs
Secondary atom site location: difference Fourier map	Flack parameter: 0.02 (8)

### Special details

**Table:**

Experimental. ESI-MS: 631.5 (*M*+Na)+ (100%); ^1^H-NMR (500 MHz, CDCl_3_): 8.27–8.15 (2*H*, dd, J1 = J2 = 16.0 Hz, 2*H*-CH—Ar), 7.72–7.64 (2*H*, m, 2*H*-3-Ar), 7.47–7.43 (2*H*, m, 2*H*-6-Ar), 7.38–7.28 (4*H*, m, 4*H*-4,5-Ar), 6.66–6.48 (2*H*, dd, J1 = J2 = 16.0 Hz, 2*H*-COCH), 6.10–6.08 (1*H*, dd, J = 3.5, 7.5 Hz, H4), 5.69–5.67 (1*H*, d, J = 5.5 Hz, H-10), 3.65 (1*H*, d, J = 5.5 Hz, H11), 3.86 (1*H*, d, J = 7.5 Hz, H2), 3.22–3.21 (1*H*, d, J = 4.0 Hz, H13), 3.18–3.15 (1*H*, d, J = 15.5 Hz, H7), 2.99–2.98 (1*H*, d, J = 4.0 Hz, H13), 2.71–2.67 (1*H*, m, H-3), 2.66–2.63 (1*H*, d, J = 15.5 Hz, H7), 2.21–2.16 (1*H*, m, H-3), 2.08 (3*H*, s, H-16), 1.06 (3*H*, s, H-14), 0.89 (3*H*, s, H-15).
Geometry. All e.s.d.'s (except the e.s.d. in the dihedral angle between two l.s. planes) are estimated using the full covariance matrix. The cell e.s.d.'s are taken into account individually in the estimation of e.s.d.'s in distances, angles and torsion angles; correlations between e.s.d.'s in cell parameters are only used when they are defined by crystal symmetry. An approximate (isotropic) treatment of cell e.s.d.'s is used for estimating e.s.d.'s involving l.s. planes.
Refinement. Refinement of *F*^2^ against ALL reflections. The weighted *R*-factor *wR* and goodness of fit *S* are based on *F*^2^, conventional *R*-factors *R* are based on *F*, with *F* set to zero for negative *F*^2^. The threshold expression of *F*^2^ > σ(*F*^2^) is used only for calculating *R*-factors(gt) *etc*. and is not relevant to the choice of reflections for refinement. *R*-factors based on *F*^2^ are statistically about twice as large as those based on *F*, and *R*- factors based on ALL data will be even larger.

### Fractional atomic coordinates and isotropic or equivalent isotropic displacement parameters (Å^2^)

**Table:**

	*x*	*y*	*z*	*U*_iso_*/*U*_eq_	
Cl1	−0.06472 (18)	0.61733 (11)	0.92949 (9)	0.0927 (4)	
Cl2	0.1261 (2)	0.79377 (10)	0.29996 (11)	0.1019 (5)	
O1	1.0020 (3)	0.56786 (16)	0.48929 (17)	0.0504 (6)	
O2	0.7296 (4)	0.76494 (16)	0.56209 (17)	0.0543 (6)	
O3	0.6673 (3)	0.63093 (17)	0.72185 (16)	0.0536 (6)	
O4	0.4986 (5)	0.5146 (2)	0.7750 (3)	0.1002 (12)	
O5	0.3826 (4)	0.4871 (2)	0.24887 (18)	0.0630 (7)	
O6	0.3161 (6)	0.4193 (4)	0.1137 (3)	0.129 (2)	
N1	0.5490 (4)	0.4323 (2)	0.2564 (2)	0.0595 (8)	
C2	0.9633 (4)	0.6377 (2)	0.5546 (2)	0.0491 (8)	
H2	1.0539	0.6883	0.5547	0.059*	
C3	0.9472 (5)	0.6005 (3)	0.6506 (3)	0.0551 (9)	
H3A	0.9799	0.6481	0.6958	0.066*	
H3B	1.0285	0.5476	0.6623	0.066*	
C4	0.7444 (5)	0.5718 (3)	0.6541 (2)	0.0496 (8)	
H4	0.7331	0.5060	0.6696	0.060*	
C5	0.6473 (4)	0.5939 (2)	0.5573 (2)	0.0424 (7)	
C6	0.6787 (4)	0.5101 (2)	0.4915 (2)	0.0440 (8)	
C7	0.6125 (5)	0.5343 (2)	0.3920 (2)	0.0507 (9)	
H7A	0.4800	0.5462	0.3881	0.061*	
H7B	0.6740	0.5905	0.3742	0.061*	
C9	0.8307 (5)	0.4096 (2)	0.3412 (3)	0.0529 (9)	
C8	0.6522 (5)	0.4579 (2)	0.3274 (3)	0.0499 (8)	
C10	0.9381 (5)	0.4258 (3)	0.4174 (3)	0.0557 (9)	
H10	1.0537	0.3973	0.4234	0.067*	
C11	0.8862 (5)	0.4870 (2)	0.4945 (2)	0.0482 (8)	
H11	0.9211	0.4551	0.5523	0.058*	
C12	0.7696 (5)	0.6729 (2)	0.5303 (2)	0.0420 (7)	
C13	0.7247 (6)	0.7472 (3)	0.4653 (3)	0.0580 (10)	
H13A	0.8243	0.7723	0.4321	0.070*	
H13B	0.6044	0.7455	0.4312	0.070*	
C14	0.4438 (5)	0.6208 (3)	0.5593 (3)	0.0581 (9)	
H14A	0.4343	0.6772	0.5934	0.087*	
H14B	0.3783	0.5721	0.5879	0.087*	
H14C	0.3908	0.6298	0.4979	0.087*	
C15	0.5706 (6)	0.4250 (3)	0.5197 (3)	0.0618 (10)	
H15A	0.6037	0.3724	0.4843	0.093*	
H15B	0.4400	0.4367	0.5091	0.093*	
H15C	0.6002	0.4126	0.5835	0.093*	
C16	0.8876 (6)	0.3440 (3)	0.2685 (3)	0.0720 (12)	
H16A	1.0136	0.3247	0.2828	0.108*	
H16B	0.8776	0.3748	0.2103	0.108*	
H16C	0.8079	0.2906	0.2661	0.108*	
C17	0.5438 (6)	0.5955 (3)	0.7761 (3)	0.0633 (10)	
C18	0.4717 (6)	0.6688 (3)	0.8326 (3)	0.0615 (10)	
H18	0.5244	0.7276	0.8317	0.074*	
C19	0.3334 (6)	0.6537 (3)	0.8852 (3)	0.0608 (10)	
H19	0.2873	0.5935	0.8873	0.073*	
C20	0.2477 (6)	0.7241 (3)	0.9399 (2)	0.0585 (10)	
C21	0.3411 (7)	0.8050 (3)	0.9676 (3)	0.0729 (12)	
H21	0.4628	0.8137	0.9530	0.087*	
C22	0.2555 (9)	0.8728 (4)	1.0165 (3)	0.0877 (15)	
H22	0.3199	0.9264	1.0346	0.105*	
C23	0.0763 (10)	0.8608 (4)	1.0381 (4)	0.0973 (18)	
H23	0.0196	0.9063	1.0714	0.117*	
C24	−0.0192 (8)	0.7831 (4)	1.0113 (3)	0.0857 (15)	
H24	−0.1418	0.7759	1.0251	0.103*	
C25	0.0657 (6)	0.7153 (3)	0.9640 (3)	0.0656 (11)	
C26	0.2778 (6)	0.4734 (4)	0.1713 (3)	0.0763 (14)	
C27	0.1139 (6)	0.5335 (3)	0.1633 (3)	0.0686 (12)	
H27	0.0286	0.5240	0.1134	0.082*	
C28	0.0775 (5)	0.5998 (3)	0.2207 (3)	0.0583 (9)	
H28	0.1621	0.6087	0.2710	0.070*	
C29	−0.0846 (5)	0.6608 (3)	0.2125 (2)	0.0576 (10)	
C30	−0.2520 (6)	0.6318 (4)	0.1689 (3)	0.0698 (11)	
H30	−0.2623	0.5720	0.1454	0.084*	
C31	−0.4065 (6)	0.6916 (4)	0.1599 (4)	0.0813 (14)	
H31	−0.5183	0.6711	0.1314	0.098*	
C32	−0.3908 (8)	0.7799 (4)	0.1935 (4)	0.0892 (16)	
H32	−0.4923	0.8197	0.1870	0.107*	
C33	−0.2279 (8)	0.8105 (4)	0.2363 (3)	0.0842 (14)	
H33	−0.2183	0.8706	0.2591	0.101*	
C34	−0.0785 (6)	0.7516 (3)	0.2454 (3)	0.0672 (11)	

### Atomic displacement parameters (Å^2^)

**Table:**

	*U*^11^	*U*^22^	*U*^33^	*U*^12^	*U*^13^	*U*^23^
Cl1	0.0732 (8)	0.1191 (11)	0.0855 (8)	−0.0194 (8)	0.0046 (6)	0.0097 (8)
Cl2	0.0834 (9)	0.1012 (10)	0.1193 (11)	−0.0005 (7)	−0.0035 (7)	−0.0454 (9)
O1	0.0376 (13)	0.0466 (13)	0.0668 (15)	−0.0018 (10)	0.0037 (10)	−0.0122 (12)
O2	0.0618 (16)	0.0385 (12)	0.0630 (15)	0.0037 (11)	0.0075 (12)	−0.0072 (11)
O3	0.0580 (15)	0.0511 (14)	0.0526 (13)	−0.0015 (12)	0.0095 (11)	−0.0035 (12)
O4	0.128 (3)	0.059 (2)	0.122 (3)	−0.014 (2)	0.064 (2)	0.0020 (19)
O5	0.0541 (16)	0.0694 (17)	0.0616 (15)	0.0174 (14)	−0.0214 (12)	−0.0176 (13)
O6	0.114 (3)	0.152 (3)	0.114 (3)	0.059 (2)	−0.038 (2)	−0.073 (2)
N1	0.0535 (19)	0.0534 (17)	0.068 (2)	0.0142 (15)	−0.0163 (15)	−0.0115 (16)
C2	0.0325 (17)	0.0462 (18)	0.069 (2)	−0.0056 (15)	0.0032 (15)	−0.0147 (17)
C3	0.042 (2)	0.060 (2)	0.061 (2)	0.0008 (17)	−0.0074 (16)	−0.0075 (19)
C4	0.053 (2)	0.0473 (19)	0.0482 (19)	0.0009 (16)	−0.0013 (16)	−0.0035 (16)
C5	0.0335 (16)	0.0460 (18)	0.0472 (17)	−0.0020 (14)	0.0000 (13)	−0.0008 (14)
C6	0.0365 (18)	0.0396 (17)	0.0544 (19)	−0.0002 (14)	−0.0064 (14)	0.0004 (15)
C7	0.046 (2)	0.0465 (19)	0.058 (2)	0.0080 (15)	−0.0101 (16)	−0.0104 (17)
C9	0.052 (2)	0.0402 (18)	0.065 (2)	0.0086 (16)	−0.0057 (17)	−0.0101 (17)
C8	0.049 (2)	0.0427 (18)	0.056 (2)	0.0040 (15)	−0.0092 (16)	−0.0032 (16)
C10	0.049 (2)	0.0450 (19)	0.070 (2)	0.0134 (17)	−0.0114 (18)	−0.0118 (19)
C11	0.0438 (19)	0.0410 (18)	0.058 (2)	0.0047 (15)	−0.0089 (15)	−0.0017 (16)
C12	0.0410 (19)	0.0313 (15)	0.0537 (19)	0.0006 (13)	0.0028 (14)	−0.0070 (15)
C13	0.069 (3)	0.049 (2)	0.056 (2)	0.0059 (18)	0.0074 (18)	0.0017 (18)
C14	0.0392 (19)	0.069 (2)	0.066 (2)	0.0027 (19)	0.0039 (16)	−0.007 (2)
C15	0.061 (2)	0.050 (2)	0.073 (3)	−0.0163 (19)	−0.0029 (19)	0.004 (2)
C16	0.076 (3)	0.058 (2)	0.079 (3)	0.020 (2)	−0.010 (2)	−0.019 (2)
C17	0.071 (3)	0.060 (2)	0.060 (2)	−0.005 (2)	0.016 (2)	0.006 (2)
C18	0.066 (3)	0.063 (2)	0.056 (2)	0.002 (2)	0.0101 (19)	−0.001 (2)
C19	0.061 (2)	0.064 (2)	0.059 (2)	−0.001 (2)	0.0131 (18)	0.0076 (19)
C20	0.063 (3)	0.068 (2)	0.045 (2)	0.005 (2)	0.0067 (18)	0.0137 (19)
C21	0.080 (3)	0.072 (3)	0.068 (3)	−0.002 (3)	0.012 (2)	0.010 (2)
C22	0.118 (5)	0.069 (3)	0.078 (3)	0.003 (3)	0.021 (3)	0.003 (3)
C23	0.131 (5)	0.081 (4)	0.085 (4)	0.019 (4)	0.046 (3)	0.012 (3)
C24	0.083 (3)	0.098 (4)	0.080 (3)	0.023 (3)	0.031 (3)	0.018 (3)
C25	0.066 (3)	0.078 (3)	0.053 (2)	0.006 (2)	0.008 (2)	0.016 (2)
C26	0.063 (3)	0.094 (3)	0.068 (3)	0.027 (2)	−0.021 (2)	−0.031 (3)
C27	0.055 (2)	0.086 (3)	0.061 (2)	0.016 (2)	−0.0186 (19)	−0.016 (2)
C28	0.047 (2)	0.072 (3)	0.054 (2)	0.0062 (19)	−0.0057 (16)	−0.0058 (19)
C29	0.051 (2)	0.072 (3)	0.0490 (19)	0.0049 (19)	0.0008 (16)	0.0027 (19)
C30	0.057 (2)	0.083 (3)	0.068 (2)	0.001 (2)	−0.0038 (19)	0.016 (2)
C31	0.052 (3)	0.105 (4)	0.086 (3)	0.010 (3)	0.000 (2)	0.030 (3)
C32	0.074 (4)	0.107 (5)	0.088 (3)	0.029 (3)	0.019 (3)	0.023 (3)
C33	0.083 (4)	0.094 (4)	0.077 (3)	0.029 (3)	0.017 (3)	0.001 (3)
C34	0.068 (3)	0.076 (3)	0.058 (2)	0.013 (2)	0.007 (2)	−0.005 (2)

### Geometric parameters (Å, °)

**Table:**

Cl1—C25	1.750 (5)	C14—H14A	0.9600
Cl2—C34	1.736 (5)	C14—H14B	0.9600
O1—C2	1.432 (4)	C14—H14C	0.9600
O1—C11	1.441 (4)	C15—H15A	0.9600
O2—C13	1.440 (4)	C15—H15B	0.9600
O2—C12	1.442 (4)	C15—H15C	0.9600
O3—C17	1.342 (5)	C16—H16A	0.9600
O3—C4	1.451 (4)	C16—H16B	0.9600
O4—C17	1.210 (5)	C16—H16C	0.9600
O5—C26	1.332 (4)	C17—C18	1.463 (6)
O5—N1	1.437 (4)	C18—C19	1.327 (5)
O6—C26	1.197 (5)	C18—H18	0.9300
N1—C8	1.286 (4)	C19—C20	1.461 (6)
C2—C12	1.506 (5)	C19—H19	0.9300
C2—C3	1.520 (5)	C20—C21	1.392 (6)
C2—H2	0.9800	C20—C25	1.394 (6)
C3—C4	1.528 (5)	C21—C22	1.385 (7)
C3—H3A	0.9700	C21—H21	0.9300
C3—H3B	0.9700	C22—C23	1.368 (8)
C4—C5	1.568 (5)	C22—H22	0.9300
C4—H4	0.9800	C23—C24	1.358 (8)
C5—C12	1.512 (4)	C23—H23	0.9300
C5—C14	1.523 (5)	C24—C25	1.371 (6)
C5—C6	1.574 (5)	C24—H24	0.9300
C6—C15	1.527 (5)	C26—C27	1.465 (6)
C6—C11	1.534 (5)	C27—C28	1.313 (5)
C6—C7	1.540 (5)	C27—H27	0.9300
C7—C8	1.495 (5)	C28—C29	1.462 (5)
C7—H7A	0.9700	C28—H28	0.9300
C7—H7B	0.9700	C29—C30	1.389 (6)
C9—C10	1.332 (5)	C29—C34	1.394 (6)
C9—C8	1.467 (5)	C30—C31	1.408 (6)
C9—C16	1.505 (5)	C30—H30	0.9300
C10—C11	1.503 (5)	C31—C32	1.365 (8)
C10—H10	0.9300	C31—H31	0.9300
C11—H11	0.9800	C32—C33	1.364 (8)
C12—C13	1.453 (5)	C32—H32	0.9300
C13—H13A	0.9700	C33—C34	1.371 (6)
C13—H13B	0.9700	C33—H33	0.9300
			
C2—O1—C11	112.9 (3)	H14A—C14—H14B	109.5
C13—O2—C12	60.6 (2)	C5—C14—H14C	109.5
C17—O3—C4	119.4 (3)	H14A—C14—H14C	109.5
C26—O5—N1	113.7 (3)	H14B—C14—H14C	109.5
C8—N1—O5	109.5 (3)	C6—C15—H15A	109.5
O1—C2—C12	107.7 (3)	C6—C15—H15B	109.5
O1—C2—C3	113.9 (3)	H15A—C15—H15B	109.5
C12—C2—C3	101.6 (3)	C6—C15—H15C	109.5
O1—C2—H2	111.1	H15A—C15—H15C	109.5
C12—C2—H2	111.1	H15B—C15—H15C	109.5
C3—C2—H2	111.1	C9—C16—H16A	109.5
C2—C3—C4	105.6 (3)	C9—C16—H16B	109.5
C2—C3—H3A	110.6	H16A—C16—H16B	109.5
C4—C3—H3A	110.6	C9—C16—H16C	109.5
C2—C3—H3B	110.6	H16A—C16—H16C	109.5
C4—C3—H3B	110.6	H16B—C16—H16C	109.5
H3A—C3—H3B	108.8	O4—C17—O3	123.4 (4)
O3—C4—C3	106.5 (3)	O4—C17—C18	126.5 (4)
O3—C4—C5	109.4 (3)	O3—C17—C18	110.1 (3)
C3—C4—C5	106.2 (3)	C19—C18—C17	121.9 (4)
O3—C4—H4	111.5	C19—C18—H18	119.1
C3—C4—H4	111.5	C17—C18—H18	119.1
C5—C4—H4	111.5	C18—C19—C20	125.2 (4)
C12—C5—C14	113.5 (3)	C18—C19—H19	117.4
C12—C5—C4	99.2 (3)	C20—C19—H19	117.4
C14—C5—C4	113.6 (3)	C21—C20—C25	116.6 (4)
C12—C5—C6	107.5 (2)	C21—C20—C19	121.7 (4)
C14—C5—C6	113.1 (3)	C25—C20—C19	121.6 (4)
C4—C5—C6	108.9 (3)	C22—C21—C20	121.0 (5)
C15—C6—C11	109.7 (3)	C22—C21—H21	119.5
C15—C6—C7	108.1 (3)	C20—C21—H21	119.5
C11—C6—C7	108.0 (3)	C23—C22—C21	120.0 (5)
C15—C6—C5	110.4 (3)	C23—C22—H22	120.0
C11—C6—C5	109.7 (3)	C21—C22—H22	120.0
C7—C6—C5	111.0 (3)	C24—C23—C22	120.4 (5)
C8—C7—C6	111.9 (3)	C24—C23—H23	119.8
C8—C7—H7A	109.2	C22—C23—H23	119.8
C6—C7—H7A	109.2	C23—C24—C25	119.8 (5)
C8—C7—H7B	109.2	C23—C24—H24	120.1
C6—C7—H7B	109.2	C25—C24—H24	120.1
H7A—C7—H7B	107.9	C24—C25—C20	122.1 (5)
C10—C9—C8	118.9 (3)	C24—C25—Cl1	117.9 (4)
C10—C9—C16	122.1 (3)	C20—C25—Cl1	119.9 (4)
C8—C9—C16	119.0 (3)	O6—C26—O5	123.5 (4)
N1—C8—C9	115.2 (3)	O6—C26—C27	123.9 (4)
N1—C8—C7	126.6 (3)	O5—C26—C27	112.6 (4)
C9—C8—C7	118.1 (3)	C28—C27—C26	125.4 (4)
C9—C10—C11	124.8 (3)	C28—C27—H27	117.3
C9—C10—H10	117.6	C26—C27—H27	117.3
C11—C10—H10	117.6	C27—C28—C29	125.7 (4)
O1—C11—C10	104.7 (3)	C27—C28—H28	117.2
O1—C11—C6	113.2 (3)	C29—C28—H28	117.2
C10—C11—C6	114.0 (3)	C30—C29—C34	116.4 (4)
O1—C11—H11	108.2	C30—C29—C28	121.5 (4)
C10—C11—H11	108.2	C34—C29—C28	122.1 (4)
C6—C11—H11	108.2	C29—C30—C31	121.1 (5)
O2—C12—C13	59.6 (2)	C29—C30—H30	119.5
O2—C12—C2	116.0 (3)	C31—C30—H30	119.5
C13—C12—C2	124.0 (3)	C32—C31—C30	119.5 (5)
O2—C12—C5	118.1 (3)	C32—C31—H31	120.2
C13—C12—C5	128.3 (3)	C30—C31—H31	120.2
C2—C12—C5	103.7 (3)	C33—C32—C31	120.7 (5)
O2—C13—C12	59.8 (2)	C33—C32—H32	119.6
O2—C13—H13A	117.8	C31—C32—H32	119.6
C12—C13—H13A	117.8	C32—C33—C34	119.4 (5)
O2—C13—H13B	117.8	C32—C33—H33	120.3
C12—C13—H13B	117.8	C34—C33—H33	120.3
H13A—C13—H13B	114.9	C33—C34—C29	122.8 (5)
C5—C14—H14A	109.5	C33—C34—Cl2	117.8 (4)
C5—C14—H14B	109.5	C29—C34—Cl2	119.4 (3)
			
C26—O5—N1—C8	172.1 (4)	O1—C2—C12—C13	−86.7 (4)
C11—O1—C2—C12	−65.2 (3)	C3—C2—C12—C13	153.4 (3)
C11—O1—C2—C3	46.7 (4)	O1—C2—C12—C5	72.6 (3)
O1—C2—C3—C4	−87.5 (3)	C3—C2—C12—C5	−47.4 (3)
C12—C2—C3—C4	28.0 (3)	C14—C5—C12—O2	37.1 (4)
C17—O3—C4—C3	−143.0 (3)	C4—C5—C12—O2	−83.8 (3)
C17—O3—C4—C5	102.6 (4)	C6—C5—C12—O2	162.9 (3)
C2—C3—C4—O3	−116.5 (3)	C14—C5—C12—C13	−34.9 (5)
C2—C3—C4—C5	0.1 (4)	C4—C5—C12—C13	−155.8 (3)
O3—C4—C5—C12	86.8 (3)	C6—C5—C12—C13	90.9 (4)
C3—C4—C5—C12	−27.8 (3)	C14—C5—C12—C2	167.0 (3)
O3—C4—C5—C14	−34.0 (4)	C4—C5—C12—C2	46.2 (3)
C3—C4—C5—C14	−148.6 (3)	C6—C5—C12—C2	−67.1 (3)
O3—C4—C5—C6	−161.0 (3)	C2—C12—C13—O2	−102.6 (3)
C3—C4—C5—C6	84.4 (3)	C5—C12—C13—O2	103.4 (4)
C12—C5—C6—C15	175.6 (3)	C4—O3—C17—O4	3.8 (6)
C14—C5—C6—C15	−58.3 (4)	C4—O3—C17—C18	−174.6 (3)
C4—C5—C6—C15	69.0 (3)	O4—C17—C18—C19	−5.6 (7)
C12—C5—C6—C11	54.6 (3)	O3—C17—C18—C19	172.8 (4)
C14—C5—C6—C11	−179.3 (3)	C17—C18—C19—C20	−176.8 (4)
C4—C5—C6—C11	−52.0 (3)	C18—C19—C20—C21	−22.9 (6)
C12—C5—C6—C7	−64.7 (3)	C18—C19—C20—C25	154.5 (4)
C14—C5—C6—C7	61.5 (4)	C25—C20—C21—C22	0.0 (6)
C4—C5—C6—C7	−171.2 (3)	C19—C20—C21—C22	177.5 (4)
C15—C6—C7—C8	−62.5 (4)	C20—C21—C22—C23	−0.2 (7)
C11—C6—C7—C8	56.1 (4)	C21—C22—C23—C24	−0.5 (8)
C5—C6—C7—C8	176.3 (3)	C22—C23—C24—C25	1.4 (8)
O5—N1—C8—C9	−177.8 (3)	C23—C24—C25—C20	−1.6 (7)
O5—N1—C8—C7	−1.6 (5)	C23—C24—C25—Cl1	−179.0 (4)
C10—C9—C8—N1	−173.9 (4)	C21—C20—C25—C24	0.9 (6)
C16—C9—C8—N1	6.7 (6)	C19—C20—C25—C24	−176.6 (4)
C10—C9—C8—C7	9.6 (6)	C21—C20—C25—Cl1	178.3 (3)
C16—C9—C8—C7	−169.8 (4)	C19—C20—C25—Cl1	0.8 (5)
C6—C7—C8—N1	143.3 (4)	N1—O5—C26—O6	1.4 (7)
C6—C7—C8—C9	−40.6 (5)	N1—O5—C26—C27	−177.1 (4)
C8—C9—C10—C11	3.5 (6)	O6—C26—C27—C28	−172.9 (6)
C16—C9—C10—C11	−177.1 (4)	O5—C26—C27—C28	5.6 (7)
C2—O1—C11—C10	176.1 (3)	C26—C27—C28—C29	179.2 (4)
C2—O1—C11—C6	51.4 (4)	C27—C28—C29—C30	28.8 (6)
C9—C10—C11—O1	−108.8 (4)	C27—C28—C29—C34	−149.4 (4)
C9—C10—C11—C6	15.4 (5)	C34—C29—C30—C31	−0.6 (6)
C15—C6—C11—O1	−166.7 (3)	C28—C29—C30—C31	−178.9 (4)
C7—C6—C11—O1	75.8 (3)	C29—C30—C31—C32	0.9 (7)
C5—C6—C11—O1	−45.3 (4)	C30—C31—C32—C33	−0.6 (7)
C15—C6—C11—C10	73.8 (4)	C31—C32—C33—C34	0.1 (8)
C7—C6—C11—C10	−43.8 (4)	C32—C33—C34—C29	0.2 (7)
C5—C6—C11—C10	−164.8 (3)	C32—C33—C34—Cl2	179.7 (4)
C13—O2—C12—C2	115.8 (4)	C30—C29—C34—C33	0.1 (6)
C13—O2—C12—C5	−120.1 (4)	C28—C29—C34—C33	178.4 (4)
O1—C2—C12—O2	−156.3 (3)	C30—C29—C34—Cl2	−179.5 (3)
C3—C2—C12—O2	83.8 (3)	C28—C29—C34—Cl2	−1.2 (6)
